# In vitro and in vivo α-amylase and α-glucosidase inhibiting activities of the protein extracts from two varieties of bitter gourd (*Momordica charantia* L.)

**DOI:** 10.1186/s12906-016-1085-1

**Published:** 2016-07-18

**Authors:** Sundar Poovitha, Madasamy Parani

**Affiliations:** Genomics Laboratory, Department of Genetic Engineering, SRM University, Kattankulathur, Chennai, 603203 India

**Keywords:** α-amylase, α-glucosidase, *Momordica charantia*, Competitive inhibition, Peak blood glucose

## Abstract

**Background:**

α-amylase and α-glucosidase digest the carbohydrates and increase the postprandial glucose level in diabetic patients. Inhibiting the activity of these two enzymes can control postprandial hyperglycemia, and reduce the risk of developing diabetes. Bitter gourd or balsam pear is one of the important medicinal plants used for controlling postprandial hyperglycemia in diabetes patients. However, there is limited information available on the presence of α-amylase and α-glucosidase inhibiting compounds. In the current study, the protein extracts from the fruits of *M. charantia* var. *charantia* (MCC) and *M. charantia* var. *muricata* (MCM) were tested for α-amylase and α-glucosidase inhibiting activities in vitro, and glucose lowering activity after oral administration in vivo.

**Results:**

The protein extract from both MCC and MCM inhibited the activity of α-amylase and α-glucosidase through competitive inhibition, which was on par with Acarbose as indicated by in vitro percentage of inhibition (66 to 69 %) and IC50 (0.26 to 0.29 mg/ml). Both the protein extracts significantly reduced peak blood glucose and area under the curve in Streptozotocin-induced diabetic rats, which were orally challenged with starch and sucrose.

**Conclusions:**

Protein extracts from the fruits of the two varieties of bitter gourd inhibited α-amylase and α-glucosidase in vitro and lowered the blood glucose level in vivo on par with Acarbose when orally administrated to Streptozotocin-induced diabetic rats. Further studies on mechanism of action and methods of safe and biologically active delivery will help to develop an anti-diabetic oral protein drug from these plants.

## Background

In diabetes mellitus, homeostasis of carbohydrate and lipid metabolism is altered due to defects in insulin production or action. It is a major non-communicable metabolic disease involving huge healthcare cost and high mortality rate. The number of adults with diabetes was estimated to be 387 million, and diabetes alone caused 4.9 million deaths in the year 2014 [1]. Postprandial hyperglycemia (PPHG) is a condition in which blood glucose level remains high after consuming meal, and it is an important factor to be considered in the management of diabetes mellitus and diabetes related secondary complications such as diabetic retinopathy, diabetic neuropathy, cardiovascular diseases, etc. [2]. Glycosidic linkages of α-D-(1,4) in carbohydrates are cleaved by α-amylase to produce oligosaccharides, which are further cleaved to monosaccharide glucose by α-glucosidase [3]. Therefore, inhibitors of these enzymes can delay the increase in blood glucose level in people who consume carbohydrate-rich food, and keep the PPHG under control [4].

Acarbose, Miglitol, and Voglibose are the enzyme inhibitors that are currently used for controlling PPHG. Acarbose inhibits both α-amylase and α-glucosidase, but Miglitol and Voglibose inhibit only α-glucosidase. Though effective in controlling PPHG, these inhibitors are not desirable for long-term treatment due to their gastrointestinal side effects [5, 6]. Given the fact that about 80 % of the diabetic people are living in low and middle income countries [7], these drugs are expensive also. Therefore, several groups have made their efforts to find α-amylase and α-glucosidase inhibitors from plants, bacteria, marine algae, and fungi [8–11]. Majority of them have studied the crude extracts (organic or aqueous), and some have studied pure compounds also [12, 13]. Most of the plant extracts and pure compounds were effective against either α-amylase or α-glucosidase, with a few exceptions being effective against both enzymes [14, 15].

Presence of antidiabetic activity in *Momordica charantia* (bitter gourd or balsam pear) was identified as early as in 1963 [16]. Extracts from fruit pulp, seeds, leaves and whole plants of *M. charantia* were shown to have hypoglycemic effects [17]. Methanol extracts from the fruits and seeds of *M. charantia* exhibited α-glucosidase inhibiting activity [18–20]. Fasting and postprandial blood glucose levels in diabetes patients were reduced after the oral intake of the aqueous extract from *M. charantia* fruit pulp [21]. Clinical trials using an insulin-like protein from *M. charantia* fruit pulp showed hypoglycemic activity in diabetes patients [22]. In vivo hypoglycemic, insulin-mimetic, and insulin secretagogue activities were also reported for the protein extracts from *M. charantia* [23, 24]. However, there was no direct evidence to show that the protein extracts from *M. charantia* have α-amylase and α-glucosidase inhibiting activities. Therefore, the current study was undertaken to evaluate the protein extracts from the fruits of two varieties of *M. charantia* for α-amylase and α-glucosidase inhibiting activities in vitro and glucose lowering activity in vivo using Acarbose as reference.

## Methods

### Materials

The fruits of *M. charantia* var. *charantia* (MCC) and *M. charantia* var. *muricata* (MCM) were bought from the local market in Chengalpet, Tamil Nadu, India. They were taxonomically identified by a botanist and verified by DNA barcoding. Porcine α-amylase and yeast α-glucosidase were bought from Sigma Aldrich, and Acarbose from Bayer AG (Germany).

### Protein extraction

Proteins were extracted from the fruit pulp of the two varieties of *M. charantia* as described before [24] with minor modifications. Fresh pulp was ground with ice-cold acid-ethanol, filtered through a muslin cloth, and centrifuged at 8000 × g for 10 min. The pH of the supernatant was adjusted to 3.0 using ammonia solution. Four volumes of acetone was added, mixed gently, and incubated at 4 °C for 24 h. The mixture was centrifuged at 6000 × g for 10 min. The pellet was washed with 80 % acetone, air dried, and dissolved in 10 mM Tris–HCl, pH 8.0.

### α-amylase inhibition assay

Stock solutions of protein extracts and Acarbose were prepared in water. Inhibition of porcine α-amylase activity was determined using dinitrosalicylic acid as described before [25]. Protein extract or Acarbose (100 μl of 2 to 20 mg/ml) was added to 100 μl of α-amylase (1 U/ml) and 200 μl of sodium phosphate buffer (20 mM, pH 6.9) to get 0.5 to 5.0 mg/ml final concentration. The samples were pre-incubated at 25 °C for 10 min, and 200 μl of 1 % starch prepared in 20 mM sodium phosphate buffer (pH 6.9) was added. The reaction mixtures were incubated at 25 °C for 10 min. The reactions were stopped by incubating the mixture in a boiling water bath for 5 min after adding 1 ml of dinitrosalicylic acid. The reaction mixtures were cooled to room temperature, diluted to 1:5 ratio with water, and absorbance was measured in a spectrophotometer (Amersham Biosciences, USA) at 540 nm. Percentage of inhibition of enzyme activity was calculated as

$$ \%\ \mathrm{Inhibition} = \left[\left({{\mathrm{A}}_{540}}^{\mathrm{Control}}\hbox{--}\ {{\mathrm{A}}_{540}}^{\mathrm{Treatment}}\right)/{{\mathrm{A}}_{540}}^{\mathrm{Control}}\right]\ \mathrm{x}\ 100 $$

wherein A_540_^Control^ is absorbance at 540 nm in control sample without protein extract and A_540_^Treatment^ is absorbance at 540 nm in treatment with protein extract

### α-glucosidase inhibition assay

Inhibition of α-glucosidase activity was determined using yeast α-glucosidase and p-nitrophenyl-α-D-glucopyranoside (pNPG) as described before [26]. Protein extract or Acarbose (100 μl of 2 to 20 mg/ml) was added to 50 μl of α-glucosidase (1 U/ml) prepared in 0.1 M phosphate buffer (pH 6.9), and 250 μl of 0.1 M phosphate buffer to get 0.5 to 5.0 mg/ml final concentration. The mixture was pre-incubated at 37 °C for 20 min. After pre-incubation, 10 μl of 10 mM pNPG prepared in 0.1 M phosphate buffer (pH 6.9) was added, and incubated at 37 °C for 30 min. The reactions were stopped by adding 650 μl of 1 M sodium carbonate, and the absorbance was measured in a spectrophotometer (Amersham Biosciences, USA) at 405 nm. Percentage of inhibition of enzyme activity was calculated as

$$ \%\ \mathrm{Inhibition} = \left[\left({{\mathrm{A}}_{405}}^{\mathrm{Control}}\hbox{--}\ {{\mathrm{A}}_{405}}^{\mathrm{Treatment}}\right)/{{\mathrm{A}}_{405}}^{\mathrm{Control}}\right]\ \mathrm{x}\ 100 $$

wherein A_405_^Control^ is absorbance at 405 nm in control sample without protein extract and A_405_^Treatment^ is absorbance at 405 nm in treatment with protein extract

### Analysis of proteolytic activity

Proteolytic activity of the plant extracts was tested against α-amylase (2 U/ml), α-glucosidase (0.05 U/ml) and a mixture of six unrelated proteins (β-lactalbumin, lysozyme, soybean trypsin inhibitor, ovalbumin, bovine serum albumin, and phosphorylase-b, 5 μg). These samples were treated with 20 μg of the protein extracts from MCC and MCM for 10 min at 25 °C (α-amylase and mixture of six proteins) or 37 °C (α-glucosidase). Treatment with Proteinase K (55 °C for 1 h), and heat denatured protein extracts were used as positive and negative controls, respectively. The reactions were stopped by heating the samples at 100 °C for 5 min after adding protein loading dye to a final concentration of 1X. The samples were analyzed by 12 % SDS-PAGE.

### Mode of inhibition assay

Mode of inhibition of α-amylase and α-glucosidase by the protein extracts was determined as described before [27]. For α-amylase, the enzyme solution (1 U/ml) was pre-incubated with protein extracts (10 mg/ml), Acarbose (10 mg/ml) or phosphate buffer (pH 6.9) at 25 °C for 10 min. The reactions were started by adding 5 to 25 mg/ml starch, and continued at 25 °C for 10 min. The reactions were stopped by adding 0.5 ml of dinitrosalicylic acid followed by boiling for 5 min. For α-glucosidase, the enzyme solution (1 U/ml) was pre-incubated with protein extracts (10 mg/ml), Acarbose (10 mg/ml) or phosphate buffer (pH 6.9) at 25 °C for 10 min. The reactions were started by adding 50 to 250 mg/ml pNPG, and continued at 25 °C for 10 min. The reactions were stopped by adding 0.05 ml of 1 M sodium carbonate. Release of reducing sugars was quantified using maltose and paranitrophenol standard curve. Double reciprocal plot (1/*v* versus 1/[S]) where *v* is reaction velocity and [S] is substrate concentration was plotted*.* Mode of inhibition was determined by analysing Lineweaver-Burk plot using Michaelis-Menten kinetics.

### Induction of diabetes in Wistar rats

Forty male Wistar rats (3-months old) were purchased from King’s Institute, Chennai, and kept under 12:12 h light and dark cycle at 25 ± 2 °C. The diabetic animals were fed with high fat diet and water ad libitum throughout the treatment period of 30 days. The experimental protocols were conducted in accordance with internationally accepted principles for laboratory animal use and were approved by the Institutional Animal Ethical Committee (087/835/IAEC-2014). Diabetes was induced in 18 h fasted animals by intraperitoneal injection of 110 mg/kg nicotinamide followed by 45 mg/kg Streptozotocin (freshly dissolved in 0.1 M citrate buffer, pH 4.5). Tail bleeds were performed 7 days after injecting Streptozotocin, and animals with blood glucose concentration above 250 mg/dL were considered diabetic.

### Oral starch and sucrose tolerance tests

Oral starch and sucrose tolerance tests were performed as described before [28]. Twenty fasted diabetic and non-diabetic rats were divided into four groups of five each, and orally treated with 10 mg/kg body weight of the protein from MCC, MCM, Acarbose (positive control) or distilled water (negative control). Ten minutes after the treatment, blood glucose level was estimated (0 min), and the rats were orally administered with 3.0 g/kg starch or 4.0 g/kg sucrose. Blood glucose level (BG) was estimated 30, 60 and 120 min after the administration. Peak blood glucose (PBG) was determined by observing the blood glucose level during the above mentioned time intervals and area under the curve (AUC) was calculated using the formula given below

$$ \mathrm{A}\mathrm{U}\mathrm{C}\ \left(\mathrm{mg}/\mathrm{dL}.\ \mathrm{H}\right) = \left({\mathrm{BG}}_0 + {\mathrm{BG}}_{30}\mathrm{x}\ 0.5\right)/2 + \left({\mathrm{BG}}_{30} + {\mathrm{BG}}_{60}\mathrm{x}\ 0.5\right)/2 + \left({\mathrm{BG}}_{60} + {\mathrm{BG}}_{120}\mathrm{x}\ 1.0\right)/2 $$

wherein BG_0_ is the blood glucose level before oral administration of starch and glucose, and BG_30,_ BG_60,_ and BG_120_ are the blood glucose level 30, 60 and 120 min after the administration.

### Statistical analysis

All experiments were performed in triplicate. Means, standard errors, and standard deviations were calculated from replicates within the experiments. Statistical analysis was done by one way analysis of variance (ANOVA). Statistical significance was accepted at *P* < 0.05. IC50 was calculated using graph pad prism software.

## Results

### α -amylase inhibition assay

**Fig. 1:**
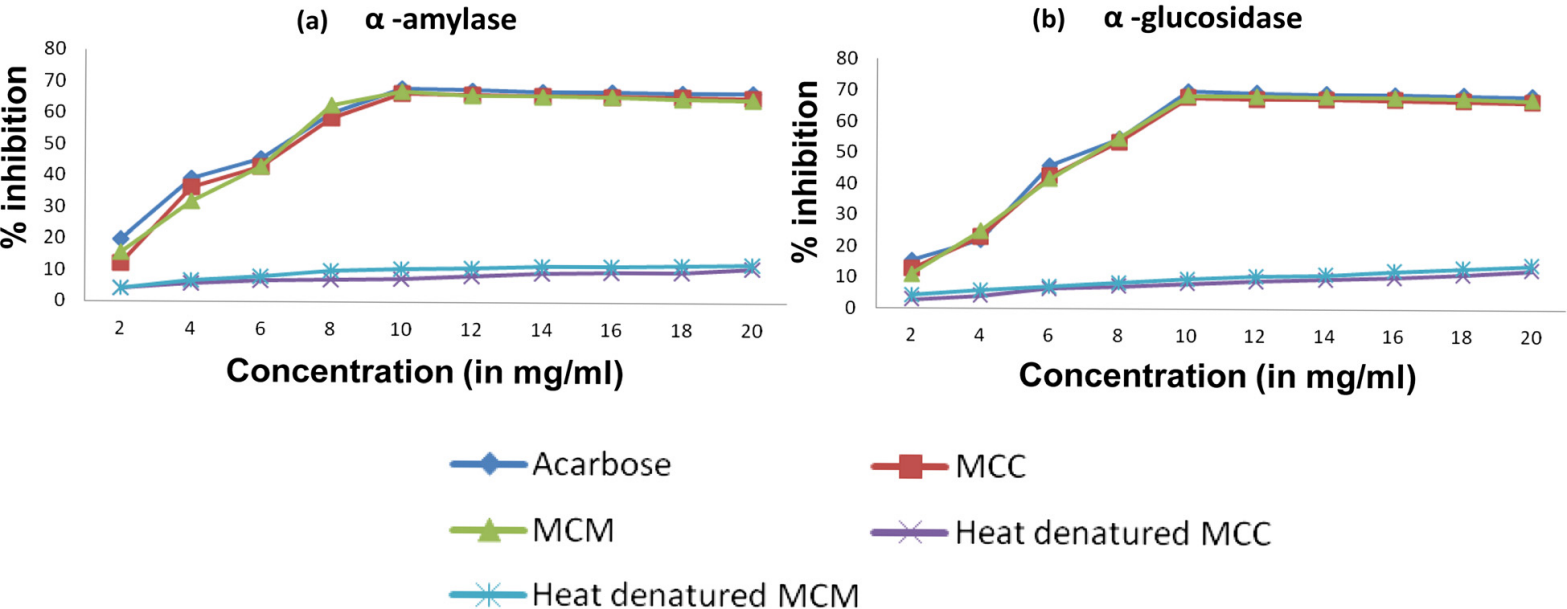
Percentage inhibition of α-amylase (**a**) and α-glucosidase (**b**) enzyme activity at different concentrations of Acarbose, protein extracts from MCC and MCM, and heat denatured protein extracts from MCC and MCM

Fruit pulp of *M. charantia* var. *charantia* (MCC) and *M. charantia* var. *muricata* (MCM) yielded 0.07 and 0.025 % proteins on wet weight basis, respectively. Inhibition of α-amylase activity by the protein extracts from MCC, MCM and Acarbose was found to be dose dependent from 0.5 to 2.5 mg/ml concentrations (Fig. 1). A maximum of 66.5, 67 and 68 % inhibition of α-amylase activity was observed at 2.5 mg/ml concentration for the protein extracts from MCC, MCM and Acarbose, respectively. Heat denatured protein extracts from MCC and MCM showed only a maximum of 11 % α-amylase inhibition activity at this concentration. The IC50 was 0.267 ± 0.024, 0.261 ± 0.019, and 0.258 ± 0.017 mg/ml for the protein extracts from MCC, MCM, and Acarbose, respectively (Table 1). The percentage inhibition of α-amylase activity and IC50 of the protein extracts from MCC and MCM was highly significant (*P* < 0.05) when compared with the heat denatured protein extracts but not with Acarbose.

**Table 1:** IC50 values of Acarbose and protein extracts from MCC and MCM for α-amylase and α-glucosidase inhibition

Analyte	IC50 (mg/ml)	
	α-amylase	α-glucosidase
Acarbose	0.258 ± 0.017	0.28 ± 0.019
MCC	0.267 ± 0.024	0.298 ± 0.034
MCM	0.261 ± 0.019	0.292 ± 0.022

### α -glucosidase inhibition assay

Ability of the protein extracts from MCC and MCM to inhibit α- glucosidase enzyme activity was determined at different concentrations between 0.5 and 5.0 mg/ml. Protein extracts from both MCC and MCM showed α-glucosidase inhibition activity in a dose dependant manner from 0.5 to 2.5 mg/ml concentration (Fig. 1). A maximum of 68.8, 69.2 and 70 % inhibition of α-glucosidase activity was observed at 2.5 mg/ml concentration for the protein extracts from MCC, MCM and Acarbose, respectively. Heat denatured protein extracts from MCC and MCM showed only a maximum of 10 % α-glucosidase inhibition activity at this concentration. The IC50 was 0.298 ± 0.034, 0.292 ± 0.022, and 0.28 ± 0.019 mg/ml for the protein extracts from MCC, MCM, and Acarbose, respectively (Table 1). The percentage inhibition of α-glucosidase activity and IC50 of protein extracts from MCC and MCM was highly significant (*P* < 0.05) when compared with heat denatured protein extracts but not with Acarbose.

### Analysis of proteolytic activity

**Fig. 2:**
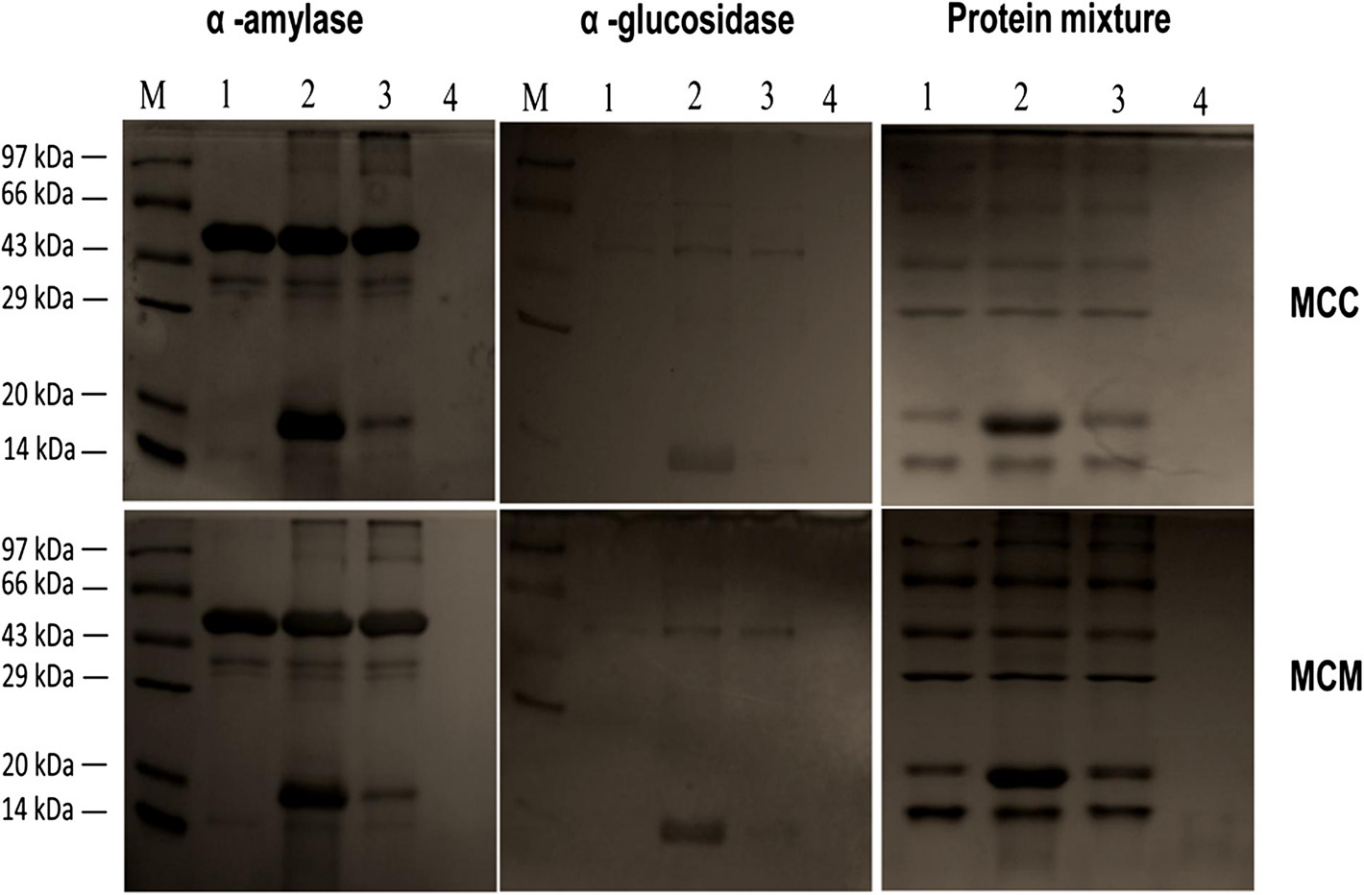
SDS-PAGE analysis of α-amylase, α-glucosidase and the protein mixture after treatment with the protein extracts from MCC and MCM. The figure shows protein marker (M), un-treated α-amylase/α-glucosidase/protein mixture (1), α-amylase/α-glucosidase/protein mixture treated with protein extracts (2) or heat denatured protein extracts (3) or Proteinase K (4)

Treatment with the protein extracts from MCC and MCM did not degrade α -amylase, α-glucosidase or the mixture of six unrelated proteins. The same result was observed when the protein extracts were heat denatured before the treatment. Treatment with Proteinase K showed complete degradation of the proteins (Fig. 2).

### Mode of inhibition assay

**Fig. 3:**
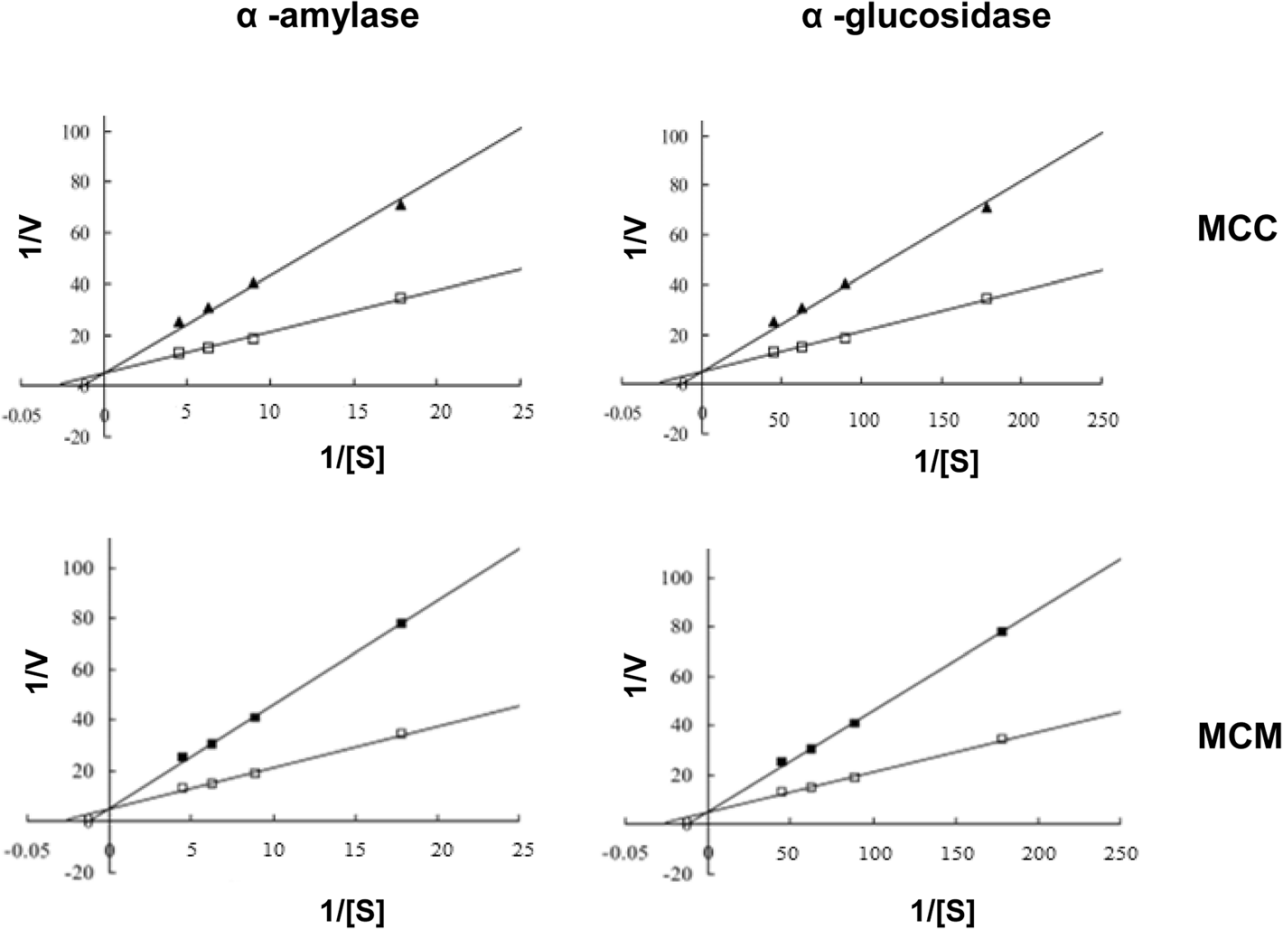
Lineweaver-Burk plots analysis of inhibition kinetics of α-amylase and α-glucosidase enzymes by the protein extracts from MCC and MCM. ▲, ■, 10 mg/ml protein extracts; □ 20 mM phosphate buffer

In the presence of the protein extracts from MCC and MCM, K_m_ increased (3.214 to 4.235 and 4.583) but V_max_ remained constant, which indicated competitive inhibition of the α-amylase enzyme activity (Fig. 3). The same mode of inhibition was observed against α-glucosidase enzyme also because K_m_ increased from 3.734 to 4.564 (MCC) and 4.789 (MCM) but V_max_ remained constant (Fig. 3).

### Oral starch and sucrose tolerance tests

**Fig. 4:**
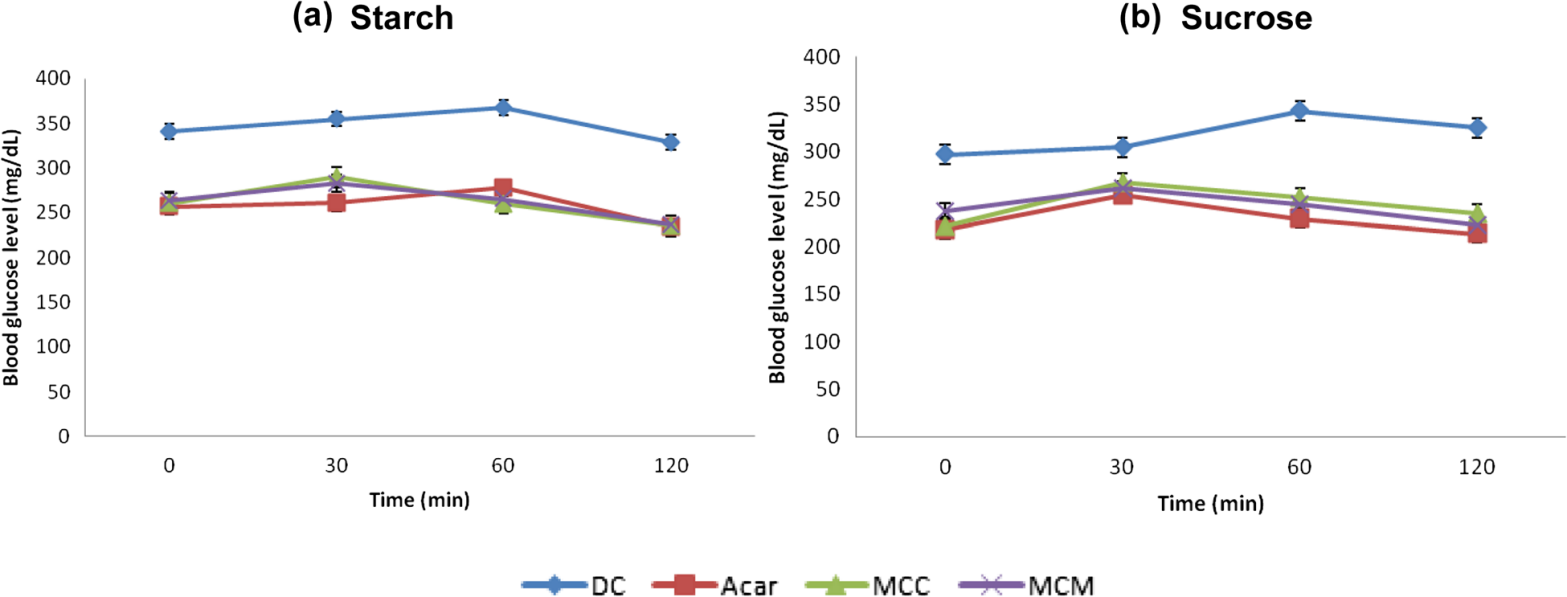
Effects of protein extracts from MCC and MCM and Acarbose on glucose concentration after the administration of 3 mg/kg starch (**a**), and 4 mg/kg sucrose (**b**) in diabetic rats. Values are the mean ± SEM (*n* = 5), at *P* < 0.05 vs. control. DC: Diabetic control, Acar: Acarbose

In both oral starch and sucrose tolerance tests, the groups that were treated with the protein extracts from MCC and MCM showed significant lowering of PBG and AUC when compared with the diabetic control group but not with the Acarbose-treated group (Fig. 4, Table 2, *P* < 0.05). In the groups that were treated with the protein extracts from MCC and MCM, the glucose level was reduced after 30 min in starch tolerance test, but only after 60 min in sucrose tolerance test. In Acarbose-treated group, the glucose level was reduced after 60 min in both the tests.

**Table 2:** Effect of Acarbose and the protein extracts from MCC and MCM on peak blood glucose (PBG) and area under the curve (AUC) after starch (3 g/kg) and sucrose (4 g/kg) loading in diabetic and non- diabetic rats. Values are the mean ± SEM (*n* = 5), at *P* < 0.05 vs. control

Group	Starch				Sucrose			
	PBG (mg/dL)	% Reduction of PBG	AUC (mg/dL)	% Reduction of AUC	PBG (mg/dL)	% Reduction of PBG	AUC (mg/dL)	% Reduction of AUC
Diabetic control	367.5 ± 0.87		702.7 ± 0.97		343 ± 0.92		646 ± 0.79	
Diabetic + Acarbose	277.8 ± 0.76	24.43	520.2 ± 0.59	25.97	254.3 ± 0.69	25.86	470 ± 0.88	27.24
Diabetic + MCC	289.8 ± 0.91	21.15	524.8 ± 0.65	25.32	267 ± 0.75	22.16	474.5 ± 0.95	26.55
Diabetic + MCM	282.8 ± 0.82	23	522.8 ± 0.78	25.6	261 ± 0.87	23.91	472 ± 0.58	26.94
Control	172 ± 0.56		382 ± 0.63		184 ± 0.81		394 ± 0.64	
Acarbose	151 ± 0.81	12.2	305.4 ± 0.67	20.05	146 ± 0.94	20.65	295.4 ± 0.56	25.03
MCC	161.2 ± 0.65	6.28	319.1 ± 0.82	16.47	153.8 ± 0.73	16.41	308.8 ± 0.52	21.62
MCM	155 ± 0.65	9.88	309.8 ± 0.56	18.9	150.2 ± 0.67	18.37	301.6 ± 0.82	23.45

## Discussion

Undesirable side effects and high cost of the currently available synthetic α-glucosidase inhibitors drive the need to explore the natural sources for new inhibitors. Being a global lifestyle disorder that affects millions of people with diverse genetic backgrounds, the search for alternate inhibitors is also desirable from the pharmacogenetics point of view. Crude extracts and purified compounds from *M. charantia* were reported to show anti-diabetic activities, which includes α-glucosidase inhibitory activity of the methanol and aqueous extracts [18–20]. However, α-amylase inhibiting activity was not reported for any extract or pure compound from this plant, and its protein extract was not studied for enzyme inhibitors against either α-amylase or α-glucosidase. Prospective inhibitors for controlling PPHG should have the ability to inhibit both α-amylase and α-glucosidase with higher percentage of inhibition and lower IC50. Therefore, protein extracts from *M. charantia* var*. charantia* (MCC) and *M. charantia* var*. muricata* (MCM) were studied for their ability to inhibit both α-amylase and α-glucosidase enzymes in vitro.

A few studies have reported that α-amylase and α-glucosidase inhibitory activity of Acarbose may range between 55 and 82 % depending on the experimental conditions [14, 15, 29]. In our study, 10 mg/ml Acarbose showed 68 and 70 % inhibition of α-amylase and α-glucosidase activity, respectively. Under the same experimental conditions, the protein extracts from both MCC and MCM showed enzyme inhibition and IC50 on par with Acarbose. To our knowledge, this is the first report of a protein extract from the same plant showing both α-amylase and α-glucosidase inhibitor activities. It remains to be studied if these two activities are contributed by a single protein or different proteins. Near complete abolition of the enzyme inhibitory activities was observed when the protein extracts from MCC and MCM were heat denatured before the treatment. This indicated that the inhibitory compounds present in the extracts may be protein in nature, though inhibition due to other thermo-labile compounds cannot be ruled out.

The protein extracts from MCC and MCM did not show proteolytic activity against α-amylase, α-glucosidase or the mixture of six unrelated proteins, which indicated the absence of proteolytic activity that is specific against these enzymes or non-specific against proteins. Therefore, the protein extracts do not contain any compound, which inhibits the activity by cleaving the enzymes. The Lineweaver-Burk plot showed that the protein extracts inhibit the α-amylase and α-glucosidase enzymes by competitive binding.

Enzyme inhibitory activity of the protein observed in vitro is often not maintained in vivo, especially in case of oral administration of protein extract which is confronted by proteolytic enzymes and harsh pH conditions. However, orally administered proteins and peptides from plants have shown significant decrease in blood glucose level indicating biologically functional activity in vivo [30, 31]. We have tested the protein extracts from MCC and MCM in vivo in Streptozotocin-induced diabetic rats. Peak blood glucose (PBG) and area under the curve (AUC) were significantly reduced in the diabetic rats that were orally administered with the protein extracts after starch or sucrose loading. The protein extracts were able to lower the blood glucose level faster than Acarbose in starch-fed diabetic rats. These results demonstrated the glucose lowering effect of the protein extracts in vivo, possibly due to the α-amylase and α-glucosidase inhibiting activities observed in vitro. However, further experiments will be needed to confirm the same or to find other possible mechanisms. Earlier studies in *M. charantia* have shown insulin-mimetic and insulin secretagogue activities in the protein extract [24], and anti-oxidant activity in the aqueous extract [32]. Therefore, the protein extract from *M. charantia* may work against diabetes through multiple mechanisms to be useful for the holistic management of diabetes mellitus.

## Conclusion

*M. charantia* is a traditional medicinal plant that is popularly used for the management of diabetes in complementary and alternative medicine, and several scientific lines of evidences were reported in favour of the same. The present study established that the protein extracts from two varieties of *M. charantia* do have α-amylase and α-glucosidase inhibiting activities in vitro and glucose lowering activity in vivo. Further research is needed to develop anti-diabetic oral protein drug from this natural source.

## Abbreviations

ANOVA, analysis of variance; AUC, area under the curve; BG, blood glucose; IC50, half maximal inhibitory concentration; MCC, *Momordica charantia* var. *charantia;* MCM, *Momordica charantia* var. *muricata;* PBG, peak blood glucose; pNPG, p-nitrophenyl-α-D-glucopyranoside; PPHG, postprandial hyperglycemia; SDS-PAGE, sodium dodecyl sulfate- polyacrylamide gel electrophoresis
